# Respiratory pathology in the TDP-43 transgenic mouse model of amyotrophic lateral sclerosis

**DOI:** 10.3389/fphys.2024.1430875

**Published:** 2024-08-27

**Authors:** Debolina D. Biswas, Ronit Sethi, Yochebed Woldeyohannes, Evelyn R. Scarrow, Léa El Haddad, Jane Lee, Mai K. ElMallah

**Affiliations:** Department of Pediatrics, Division of Pulmonary and Sleep Medicine, Duke University Medical Center, Durham, NC, United States

**Keywords:** amyotrophic lateral sclerosis, breathing abnormalities, TDP-43 (43kda TAR DNA binding protein), phrenic and hypoglossal nerve, motor neurodegeneration, neuroinflammation, respiration

## Abstract

Amyotrophic lateral sclerosis (ALS) is a devastating neurodegenerative disease that results in death within 2–5 years of diagnosis. Respiratory failure is the most common cause of death in ALS. Mutations in the transactive response DNA binding protein 43 (TDP-43) encoded by the *TARDBP* gene are associated with abnormal cellular aggregates in neurons of patients with both familial and sporadic ALS. The role of these abnormal aggregates on breathing is unclear. Since respiratory failure is a major cause of death in ALS, we sought to determine the role of TDP-43 mutations on the respiratory motor unit in the Prp-hTDP-43^A315T^ mouse model – a model that expresses human TDP-43 containing the A315T mutation. We assessed breathing using whole-body plethysmography, and investigated neuropathology in hypoglossal and phrenic respiratory motor units. Postmortem studies included quantification of hypoglossal and putative phrenic motor neurons, activated microglia and astrocytes in respiratory control centers, and assessment of hypoglossal and phrenic nerves of TDP43^A315T^ mice. The male TDP43^A315T^ mice display an early onset of rapid progression of disease, and premature death (less than 15 weeks) compared to control mice and compared to female TDP43^A315T^ mice who die between 20 and 35 weeks of age. The TDP43^A315T^ mice have progressive and profound breathing deficits at baseline and during a respiratory challenge. Histologically, hypoglossal and putative phrenic motor neurons of TDP43^A315T^ mice are decreased and have increased microglial and astrocyte activation, indicating pronounced neurodegeneration and neuroinflammation. Further, there is axonopathy and demyelination in the hypoglossal and phrenic nerve of TDP43^A315T^ mice. Thus, the TDP-43^A315T^ mice have significant respiratory pathology and neuropathology, which makes them a useful translatable model for the study of novel therapies on breathing in ALS.

## 1 Introduction

Amyotrophic Lateral Sclerosis (ALS) is a devastating and fatal neurodegenerative disease with no current cure. ALS patients experience both upper and lower motor neuron loss (Nichols et al., 2013; Brown and Al-Chalabi, 2017). As the disease progresses, motor neuron pathology causes axonal retraction from the tongue, diaphragm, and intercostal muscle fibers resulting in respiratory muscle weakness and atrophy (Nichols et al., 2013). Increasing atrophy of the diaphragm leads to dyspnea, orthopnea, and hypoventilation, while weakness of the tongue muscle, including genioglossus muscle contributes to dysarthria, dysphagia, an increased risk of aspiration pneumonia, and diminished upper airway patency (Niedermeyer et al., 2019; Vacchiano et al., 2021). As a result, most patients with ALS have progressive respiratory insufficiency which leads to respiratory failure due to impaired ventilation and aspiration pneumonia (Vitacca et al., 1997; Gil et al., 2008; Wolf et al., 2017).

Recent genomic studies in patients with both familial and sporadic ALS identified mutations in more than 40 genes associated with ALS (Smukowski et al., 2022). Mutations in the *TARDBP* gene which encodes the TAR-DNA-binding protein 43 (TDP-43) are implicated in the pathogenesis of ALS and Frontotemporal Lobar Degeneration (FTLD) (Neumann et al., 2006; Pesiridis et al., 2009). TDP-43 is a ubiquitously expressed DNA/RNA binding protein involved in RNA transcription, splicing, mRNA transport, and microRNA biosynthesis (Buratti and Baralle, 2010). Several TARDBP missense mutations lead to the gain of toxic function or loss of function in TDP-43 which causes a range of pathologies (Barmada et al., 2010; Guo et al., 2011; Tsao et al., 2012; Prasad et al., 2019; Jo et al., 2020).

Patients with the A315T mutation in TDP-43 have respiratory involvement and progressive respiratory weakness (Gitcho et al., 2008). However, the role of TDP-43-associated pathology in respiratory dysfunction remains unclear. In this study, we evaluated the respiratory function of transgenic Prp-hTDP-43^A315T^ (will be further referred to as TDP-43 ^A315T^ mice), a mouse model of ALS and age/sex-matched wildtype (WT) each month, until the TDP-43 ^A315T^ mice reached their terminal stage (Wegorzewska et al., 2009). In post-mortem studies, we focus on the hypoglossal and phrenic motor nucleus. Patients with ALS exhibit TDP-43 inclusions with loss of motor neurons in the hypoglossal nucleus, cervical spinal cord anterior horn, and cervical anterior nerve roots (Dickson et al., 2007; Brettschneider et al., 2014; Mori et al., 2014; Halliday et al., 2016; Nakamura-Shindo et al., 2020). The hypoglossal (in the medulla) and phrenic (in the C3–C5 region of the cervical spinal cord) motor units play a vital role in breathing. The hypoglossal nerve innervates the tongue to maintain its shape, stiffness, and position to regulate upper airway patency (Fuller et al., 1999; Bailey, 2011). The phrenic nerve innervates the diaphragm which is the major inspiratory muscle (Fogarty et al., 2018). Loss of respiratory motor neurons and nerves can impede breathing and result in respiratory failure in ALS (Pinto et al., 2009; Pinto et al., 2012).

Motor neuron degeneration is linked with neuroinflammation and has been presented in both ALS patients and mouse models of ALS. ALS-associated neuroinflammation is typically characterized by activation of resident glial cells such as astrocytes, microglia and other peripheral immune cells (Liu and Wang, 2017; McCauley and Baloh, 2019; Liu et al., 2021; Humphrey et al., 2023). Activation of glia such as astrocytes and microglia in ALS is critical for inducing neuroinflammation by generating excessive proinflammatory cytokines and other toxic mediators, all of which results in motor neuron degeneration. (Yamanaka et al., 2008; Frakes et al., 2014; Rojas et al., 2014; Ban et al., 2019; Tam et al., 2019; You et al., 2023). Thus, our overall goal was to study the respiratory function of the TDP-43 ^A315T^ mice and to investigate neurodegeneration and neuroinflammation in the hypoglossal and putative phrenic respiratory control centers. We also sought to assess the hypoglossal and phrenic nerves of TDP-43 ^A315T^ mice for evidence of axonopathy. Altogether our findings indicate that A315T mutation in TDP-43 contributes to respiratory insufficiency in ALS.

## 2 Method and material

### 2.1 Mice

C57BL6/J (wildtype) (JAX strain # 000664) and Prp-TDP-43^A315T^ (JAX strain # 010700) mice were obtained from the Jackson Laboratory (Wegorzewska et al., 2009). These animals were bred and housed at the Duke University Division of Laboratory Animal Resources on a 12-h light/dark cycle with *ad libitum* access to food and water. The littermates obtained from the breeding of C57BL6/J and Prp-TDP-43^A315T^ mice were genotyped and used for the experiments. These mice were provided chow and HydroGel (Clear H_2_O, Portland, ME, United States) packs to supplement their regular food and water supply from 6 weeks of age to males and from 18 weeks of age to females. Mice were observed for kyphosis, weighed, and examined for ability to right back within 15 s weekly. Mice were euthanized at a predetermined humane endpoint or “end stage”. The criteria for end-stage mice were severe kyphosis, loss of weight by 15% and loss of ability to right back within 15 s. Animal studies were conducted under approved protocols from Duke University Institutional Animal Care and Use Committee (IACUC).

### 2.2 Respiratory analysis by whole-body plethysmography

Whole-body plethysmography was performed as described before (McCall et al., 2020; Fusco et al., 2021). The weight and body temperature of the mice were recorded before the experiment. Unanesthetized and unrestrained mice were placed in a Plexiglas chamber (DSI, St. Paul, MN).

Baseline: The mice were placed in whole body plethysmography chamber at room air and monitored. These mice are allowed to be acclimatized to their surroundings for 30 min. Following the acclimation period, ventilation was further monitored under room air conditions (normoxia: FiO_2_: 0.21; N_2_ balance) for another 1.5 h. Within this timeframe, we selected 5 min in which mice were sleeping and maintained a pattern of regular breathing. This phase of breathing is selected as the “Baseline.”

Challenge: Following the period of exposure to room air, mice were then exposed to a hypercapnic and hypoxic (FiCO_2_: 0.07, FiO_2_: 0.10; N_2_ balance) respiratory challenge for 10 min. This phase of 10 min of breathing is selected as a “Challenge.” Mice were then returned to room air and observed until they returned to normal breathing.

Data was recorded and analyzed using FinePointe Software. The whole-body plethysmography was performed at different ages to detect the progression of respiratory dysfunction.

### 2.3 Immunohistochemistry of medulla and cervical spinal cord

Every sixth 20-micron section of the cervical spinal cord in the region of the putative phrenic motor nucleus and medulla in the region of the hypoglossal motor nucleus was stained for choline acetyltransferase (ChAT) to label motor neurons. The free-floating sections were placed in 96 well plates, washed in PBS thrice for 5 min, and quenched for 1 h using 0% 1X PBS, 30% Methanol, and 18% H_2_O_2_ for 1 h, Further the tissues were blocked with 10% normal horse serum for 1 h (Vector Laboratories, Burlingame, CA, United States, S-2000) and incubated overnight in primary antibody solution (1:100, polyclonal goat anti-ChAT, Millipore, Burlington, MA, United States, AB144P). The next day, the tissues were washed with 1X PBS followed by incubation in secondary antibody [1:200, biotin-SP-conjugated AffiniPure Donkey Anti-Goat IgG (H + L), Jackson ImmunoResearch, West Grove, PA, United States, 705-065-003] for 2 h. Vectastain Avidin-Biotin Complex (ABC) Kit was used (Vector Laboratories, Burlingame, CA, United States, PK-4000) was used as per the manufacturer’s protocol. In short, the tissues were incubated for 2 h in 98.7% 1x PBS, 0.5% Reagent A, 0.5% Reagent B and 0.3% Triton x-100. Then the tissues were treated with 3,3′-Diaminobenzidine (DAB) Substrate Kit (Vector Laboratories, Burlingame, CA, United States, SK-415) as per the manufacturer’s protocol for 10 min. In short, the tissues were incubated in 1 mL DAB Dilutent and 1 drop Impact DAB with 0.03% hydrogen peroxide. After 10 min, the tissues were washed and mounted on slides. Further, these tissues were dried, stained with 0.1% Cresyl Violet acetate for 2 min, washed in distilled water, and treated with 50%, 70%, 95%, and 100% ethanol for 1 min each, followed by 2 min in xylene. The coverslip was sealed with DPX mounting media on the slides. The hypoglossal and putative phrenic motor neuron counts were assessed in WT and TDP-43^A315T^ mice using ImageJ by blinded observers.

To stain for motor neurons, microglia and astrocytes, every sixth 20-micron free floating cross-sections from the regions containing the phrenic and hypoglossal motor pools in WT and TDP-43^A315T^ mice were placed in 96 well plates. The tissues were washed, quenched, blocked, and stained with primary antibodies as mentioned above on day 1. The tissues were dual stained with anti-ChAT (1:250, Millipore, Burlington, MA, United States, AB144P-1 ML) and anti-Iba1 (1:300, FUJIFILM Wako Pure Chemical Corporation, Tokyo 019-19741) to stain for motor neurons and microglia. We used anti-GFAP (1:500, EnCor Biotechnology Inc, CPCA-GFAP) to stain for astrocytes. On day 2, sections were washed and then incubated for 2 h in a secondary antibody solution consisting of 1:500 anti-goat IgG Alexa Fluor 488 (Invitrogen, Carlsbad, CA, United States, A32814),1:500 anti-rabbit IgG Alexa Fluor 594 (Invitrogen, Carlsbad, CA, United States, A32754) and 1:500 anti-chicken IgG Alexa Fluor 647 (Invitrogen, Carlsbad, CA, United States, A-21449). Sections were mounted on Vectashield Antifade Mounting Medium with DAPI (Vector Laboratories, Burlingame, CA, United States). For negative control staining, tissues were stained with secondary antibodies only.

### 2.4 Nerve processing and imaging

Phrenic and XII nerves were harvested from terminal-stage WT and TDP-43^A315T^ male mice and placed in 2.5% glutaraldehyde and 0.1% sodium cacodylate. They were then processed, embedded in hard plastic, sectioned to 1 μm, and stained with 1% toluidine blue and 1% sodium borate by the Duke University Electron Microscopy Core and were imaged on brightfield using an ECHO Revolve microscope. These images were analyzed using a public downloadable ImageJ plugin to examine the g-ratio and axonal area. The g-ratio is the measure of the ratio of the axon diameter to the diameter of the axon plus myelin and is a highly reliable indicator of optimal myelination. 100 randomly selected axons from each nerve were manually outlined for each animal.

### 2.5 Behavioral tests

Neurobehavioral assessments were performed as described previously (Guyenet S. J. et al., 2010; Fusco et al., 2021). This protocol grades the pathology in mice on a four-point scale (0–5, with 0 indicating an absence of pathology and 5 indicating the most severe pathology) in four distinct tests: a clasping test, a gait test kyphosis, a ledge test and wire hang latency test.

Ledge test: Lift the mouse from the cage and place it onto the cage ledge. Watch the mouse as it walks along the cage ledge. Scores are assigned as follows

0: If the mouse does not lose balance and lowers itself into the cage gracefully using its paws
1: If the mouse loses footing but otherwise appears coordinated
2: If the mouse does not effectively use hindlimbs or cannot gracefully lower itself into the cage
3: If the mouse falls off the ledge or nearly, experiences tremors while walking and refuses to move at all despite encouragement

Hindlimb Casping: Lift the mouse by the base of the tail and observe the hindlimb position. Scores are assigned as follows

0: If the hindlimbs are consistently displayed outward and away from the abdomen
1: If one of the hindlimbs is retracted towards the abdomen for more than 50% of the time
2: If both the hindlimbs are partially retracted towards the abdomen for more than 50% of the time
3: If both the hindlimbs are entirely retracted towards the abdomen for more than 50% of the time

Gait: The mice were positioned on a benchtop and their movement was analyzed from behind as they walked. Scores were assigned as follows

0: If the mouse moves normally with its body weight distributed on all limbs, the abdomen does not touch the ground, and both hindlimbs participate evenly
1: If the mouse shows tremors, appears to limp while walking, or feet are slightly pointed away from the body
2: If the mouse shows severe tremor, a severe limp, the pelvis is lowered and, the feet point away more severely from the body
3: If the mouse has difficulty moving forward and drags its abdomen along the ground.

Kyphosis: The spinal curvature of each mouse was analyzed when the mouse was rested and while in motion.

0: If the mouse can easily straighten the spine as it walks
1: If the mouse exhibits mild kyphosis (hump), but can straighten its spine as it walks
2: If the mouse cannot straighten the spine completely and maintains consistent, but mild kyphosis (hump)
3: Pronounced kyphosis (hump) while the mouse walks or sits.

Wire hang test: The mice were placed on an elevated wire grid, then inverted and suspended above the home cage for 120 s. The latency to when the animal falls was recorded. This test is performed with two trials per session and an average of these three trials was presented.

### 2.6 Statistical analysis

GraphPad Prism Software was used to analyze data. Data from age-dependent whole-body plethysmography, behavioral tests, and weights were analyzed using a mixed-model two-way ANOVA. repeated measures (RM) ANOVA. and *post-hoc* analysis was performed using a Fisher’s LSD test. Quantification of ChAT^+^, Iba1^+^, GFAP^+^ cells, and G-ratio of nerves were analyzed using a Student’s *t-test*. For all statistical analyses, significance is defined as *p < 0.05, **p < 0.01, ***p < 0.001, ****p < 0.0001. Data are reported as mean ± SEM.

## 3 Results

### 3.1 TDP-43^A315T^ mice have a shorter lifespan with different timelines of disease progression between males and females

**FIGURE 1:**
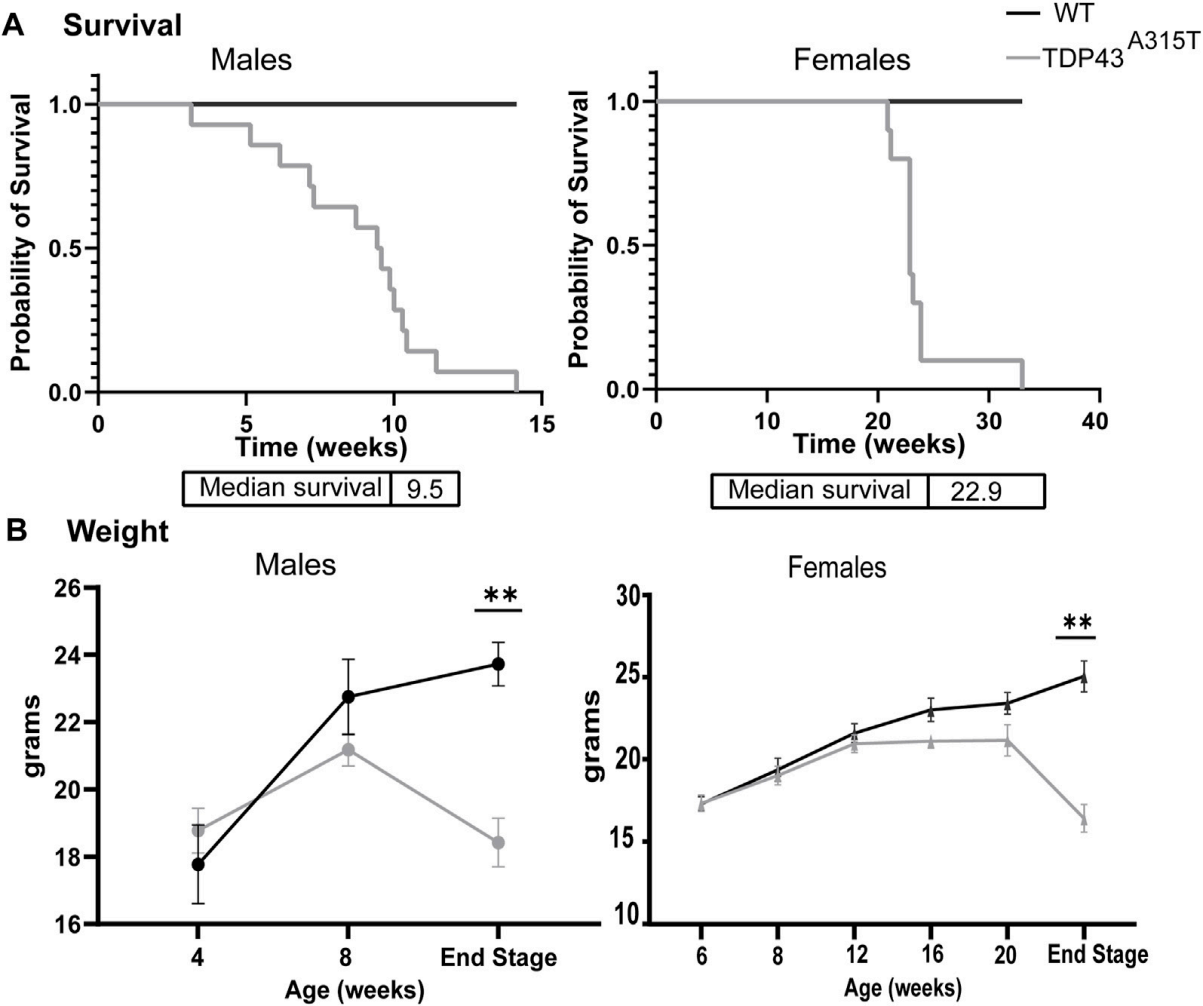
TDP-43^A315T^ mice experience premature death and significant weight loss. **(A)** Kaplan-Meier survival curve of male and female WT and TDP-43^A315T^ mice with median survival age. (n = 14 males/genotype; n = 10 females/genotype) **(B)** The weight of the male and female WT and TDP-43^A315T^ mice at indicated ages and at the end stage. Data are mean ± SEM. (n = 5 males and females/genotype). Statistical significance was determined using unpaired Student’s T-test (*P < 0.05; **P < 0.01).

TDP-43 ^A315T^ mice experienced premature death compared to their WT littermates. The female TDP-43 ^A315T^ mice survived longer than the male TDP-43 ^A315T^ mice. On average, the male TDP-43^A315T^ mice survived for about 9.5 weeks while the female Prp-TDP-43^A315T^ mice survived for about 23 weeks (Figure 1A). Both male and female Prp-TDP-43^A315T^ mice showed no symptoms at birth and appeared normal. However, the onset of the symptoms was sudden and progressed rapidly till they reached their terminal stage. This phenomenon has also been reported by others (Wegorzewska et al., 2009; Esmaeili et al., 2013; Herdewyn et al., 2014). The TDP-43^A315T^ males gained weight steadily until 8 weeks of age and showed a sudden decline until it reached its terminal stage (Figure 1B). In contrast, the female TDP-43^A315T^ mice gained weight till 12-week of age and plateaued till the 20 week of age. However, with disease progression, the females failed to thrive and lost significant weight compared to their age-matched wild-type littermates (Figure 1B). In males, the onset of symptoms is early while in females, the onset is late. However, the progression of the disease is rapid in both males and females.

### 3.2 TDP-43^A315T^ mice experience progressive respiratory insufficiency

**FIGURE 2:**
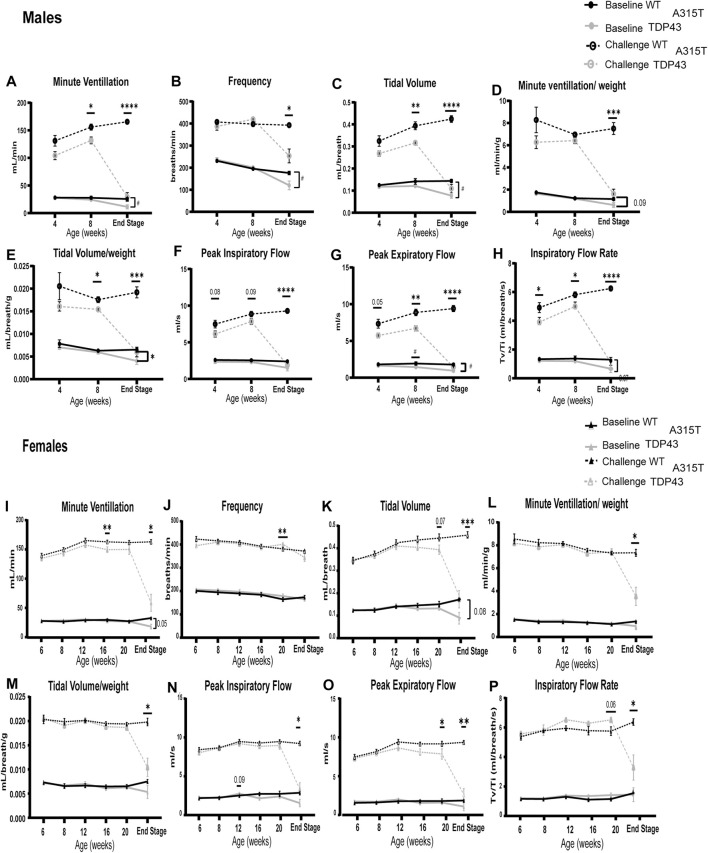
TDP-43^A315T^ mice have pronounced respiratory deficits in normoxia and hypercapnic/hypoxic conditions which exacerbate at the end stage. Measures of respiratory function in both males **(A–H)** and females **(I–P)** in WT and TDP-43 mice – Minute Ventilation **(A,I)**, frequency **(B,J)**, tidal volume **(C,K)**, Minute ventilation/weight **(D,L)**, Tidal volume/weight **(E,M)**, peak inspiratory flow **(F,N)**, peak expiratory flow **(G,O)**, Inspiratory flow rate **(H,P)** is evaluated during normoxia (“Baseline”) and a maximal respiratory challenge with hypoxia + hypercapnia. Data are mean ± SEM. Statistical significance was determined using **(A)** mixed-model two-way ANOVA, followed by a Fisher’s LSD test (“#” indicates significance during baseline; #*P* < 0.05; ##*P* < 0.01. “*” indicates significance of challenge *P < 0.05; **P < 0.01; ***P < 0.001 ****P < 0.0001). For all age groups except end-stage n = 5 WT, 7 TDP-43 ^*A315T*^ males and n = 5 WT, 7 TDP-43 females. For the end stage, n = 4 WT, 5 TDP-43 ^*A315T*^ males, and n = 5 WT, 4 TDP-43 ^*A315T*^ females.

The respiratory function of WT and TDP-43^A315T^ mice was assessed monthly at baseline (normoxia, FiO_2_: 0.21; N_2_ balance) and during a hypoxic-hypercapnic challenge (FiO_2_: 0.10; FiCO_2_: 0.07; N_2_ balance) by whole-body plethysmography (Figure 2). Age-matched males (Figures 2A–F) (n = 5 WT and 7 TDP-43^A315T^ mice) and females (Figures 2G–L) (n = 5 WT and 5 TDP-43^A315T^ mice) were examined monthly. Due to the rapid progression of symptoms and sudden death of the TDP-43^A315T^ mice, our sample size decreased for both male (n = 4) and female (n = 4) TDP-43^A315T^ mice. A reduced ventilatory response to the challenge was evident at an early age of 4 weeks in the male TDP-43^A315T^ mice. The 4-week male TDP-43^A315T^ mice showed a decreasing trend in minute ventilation (MV), tidal volume (TV), peak inspiratory flow (PIF), and peak expiratory flow (PEF), and significantly reduced inspiratory flow rate (TV/Ti) compared to age-matched WT mice. At 8 weeks of age, TDP-43^A315T^ male mice exhibit significant deficits in respiratory parameters which continue to worsen until end-stage. By end-stage, the male TDP-43^A315T^ mice show significantly reduced MV (Figure 2A; *p* = 0.0173 at baseline; *P* < 0.0001 at challenge), frequency (F) (Figure 2B; *P* = 0.0438 at baseline; *P* = 0.0190 at challenge), TV (Figure 2C; *P* = 0.0022 at baseline; *P* < 0.0001 at challenge), minute ventilation normalized to weight (Figure 2D; *p* = 0.09 at baseline; *P =* 0.0002 at challenge), tidal volume normalized to weight (Figure 2E; *P* = 0.03 at baseline; *P =* 0.0002 at challenge) PIF (Figure 2F; *P* = 0.113 at baseline; *P* = <0.0001 at challenge), and PEF (Figure 2G; *P* = 0.0134 at baseline; *P* < 0.0001 at challenge), TV/Ti (Figure 2H; *P* = 0.07 at baseline and *P* < 0.0001 at challenge) when compared to male WT age-matched littermate.

Unlike males, female TDP-43^A315T^ mice have late-onset of disease symptoms but exhibit a fast progression of disease similar to that of the male TDP-43^A315T^ mice. Their symptoms dramatically worsen as the mice reach their end-stage (at about 22–23 weeks of age). In contrast to TDP-43^A315T^ male mice, when compared to age-matched female WT littermates, we observed that the female TDP-43^A315T^ mice show a steep decline in respiratory parameters only when challenged with hypoxia and hypercapnia. The female TDP-43^A315T^ exhibits a significantly reduced MV (Figure 2I; *P* = 0.032 at challenge), TV (Figure 2K; *P* = 0.001 at challenge), minute ventilation normalized to weight (Figure 2L; *P* = 0.2 at baseline; *P* = 0.03 at challenge), tidal volume normalized to weight (Figure 2M; *P* = 0.25 at baseline; *P* = 0.02 at challenge) PIF (Figure 2N: *P* = 0.01 at challenge), PEF (Figure 2O; *P* = 0.008 at challenge), and TV/Ti (Figure 2P; *P* = 0.02 at challenge). No difference was observed in F (Figure 2J; *P* = 0.29) between female WT and TDP-43^A315T^ mice during baseline and challenge. We have normalized the tidal volumes and minute volumes to the body weight of the mice to confirm if the weight loss of the TDP-43^A315T^ mice influenced the respiratory pathology, The differences in TV normalized to weight in males were evident at 8 weeks of age and significantly reduced in TDP-43^A315T^ mice when challenged at the end stage. MV when normalized to weight in male mice was significantly reduced at endstage. Similarly, the differences in Tidal volume and minute ventilation in females WT and TDP-43^A315T^ mice were significantly different at the end stage irrespective of the weight differences.

**FIGURE 3:**
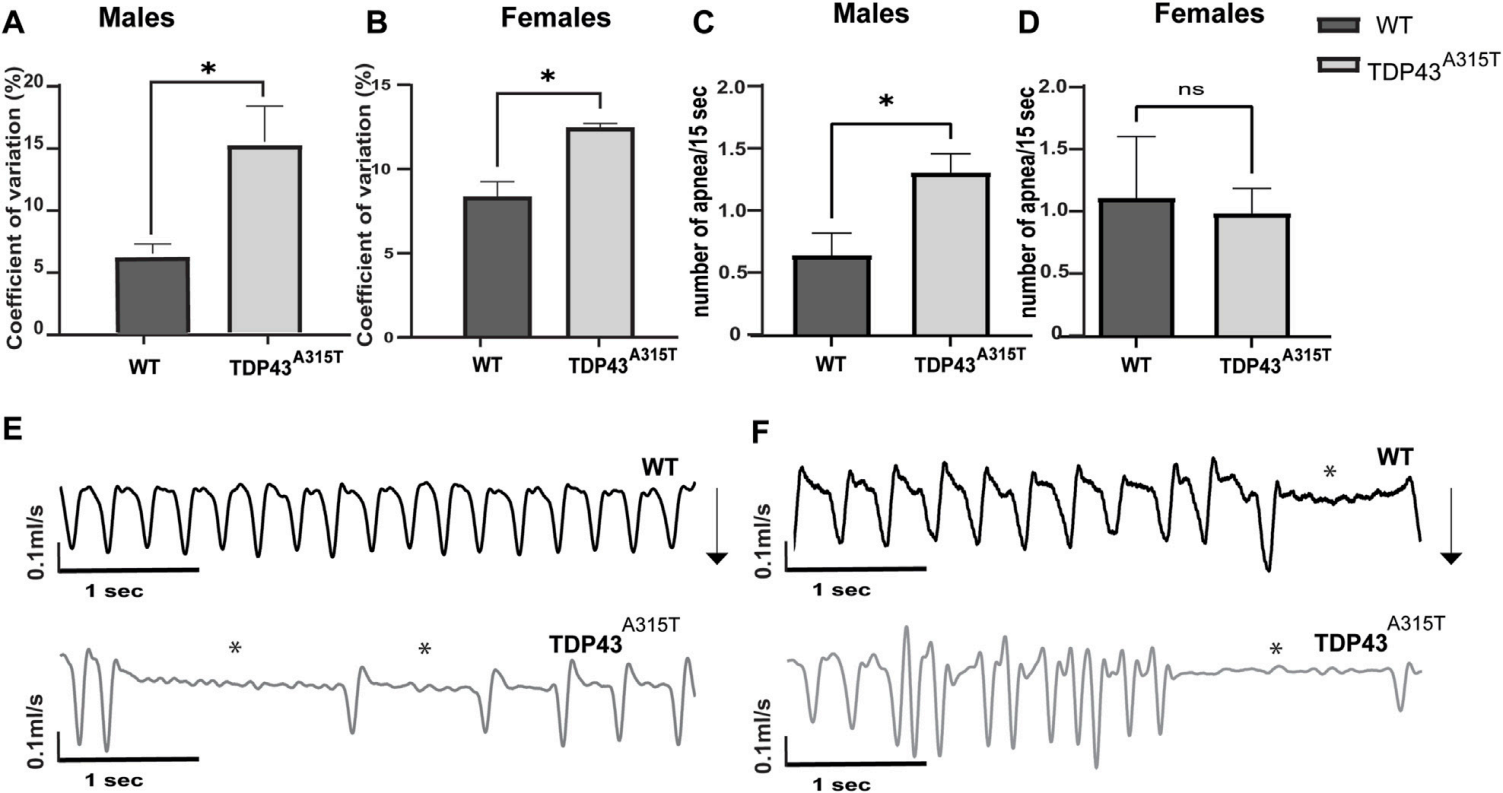
TDP-43^A315T^ mice breathe erratically with the progression of the disease **(A,B)** Graphical presentation of coefficient of variation of inspiratory time in 8 weeks male **(A)** and 20 weeks female **(B)** TDP-43 ^*A315T*^ mice compared to age and sex-matched wildtype mice at baseline. **(C,D)** Graphical representation of the number of apnea evaluated over 1 h of normoxia in 8 weeks male **(C)** and 20 weeks female **(D)** TDP-43 ^*A315T*^ mice compared to age and sex-matched wild-type mice. Data are mean ± SEM. Statistical significance was determined using unpaired Student’s T-test (*P < 0.05) (n = 3/genotype) **(E,F)** Representative tracings of wildtype and TDP-43 ^*A315T*^ male **(E)** and female **(F)** mice at baseline. Arrows indicate the direction of the inspiration in the mice.

Both male and female TDP-43^A315T^ mice have increased variability in their respiratory pattern [as calculated by the coefficient of variation (CV) for inspiratory time (Ti)] compared to wild-type mice (Figure 3). The TDP-43^A315T^ mice have increased variability in Ti at the end-stage during baseline breathing which indicates more erratic breathing. To understand if the erratic breathing preceded the end stage of the disease, we analyzed the CV in Ti of 8 weeks male and 20 weeks female WT and TDP-43^A315T^ mice. A notable increase in CV of Ti is observed in both males (Figure 3A; *P* = 0.04) and females (Figure 3C; *P* = 0.011) at baseline. ALS patients experience apnea, therefore, we assessed the apneic events in TDP-43^A315T^ mice and compared them with WT mice. Several apneic events were observed in both WT and TDP-43^A315T^ mice which had been previously documented in C57Bl6 mice (Stettner et al., 2008). Compared to the male WT controls, the TDP-43^A315T^ mice experienced an increased number of apnea (Figure 3C; *p = 0.019*) while no differences were observed in female WT and TDP-43^A315T^ mice (Figure 3D; *P =* 0.8). Representative tracings of WT and TDP-43^A315T^ males (Figure 3E) and females (Figure 3F) show variability in breathing. Episodes of apnea are evident in TDP-43^A315T^ mice at baseline (normoxia) as indicated by asterisks. Altogether, these findings indicate progressive respiratory pathology in TDP-43^A315T^ mice which is much more severe at the disease end-stage.

### 3.3 TDP-43^A315T^ mice have neurodegeneration and neuroinflammation in hypoglossal and phrenic motor nuclei

**FIGURE 4:**
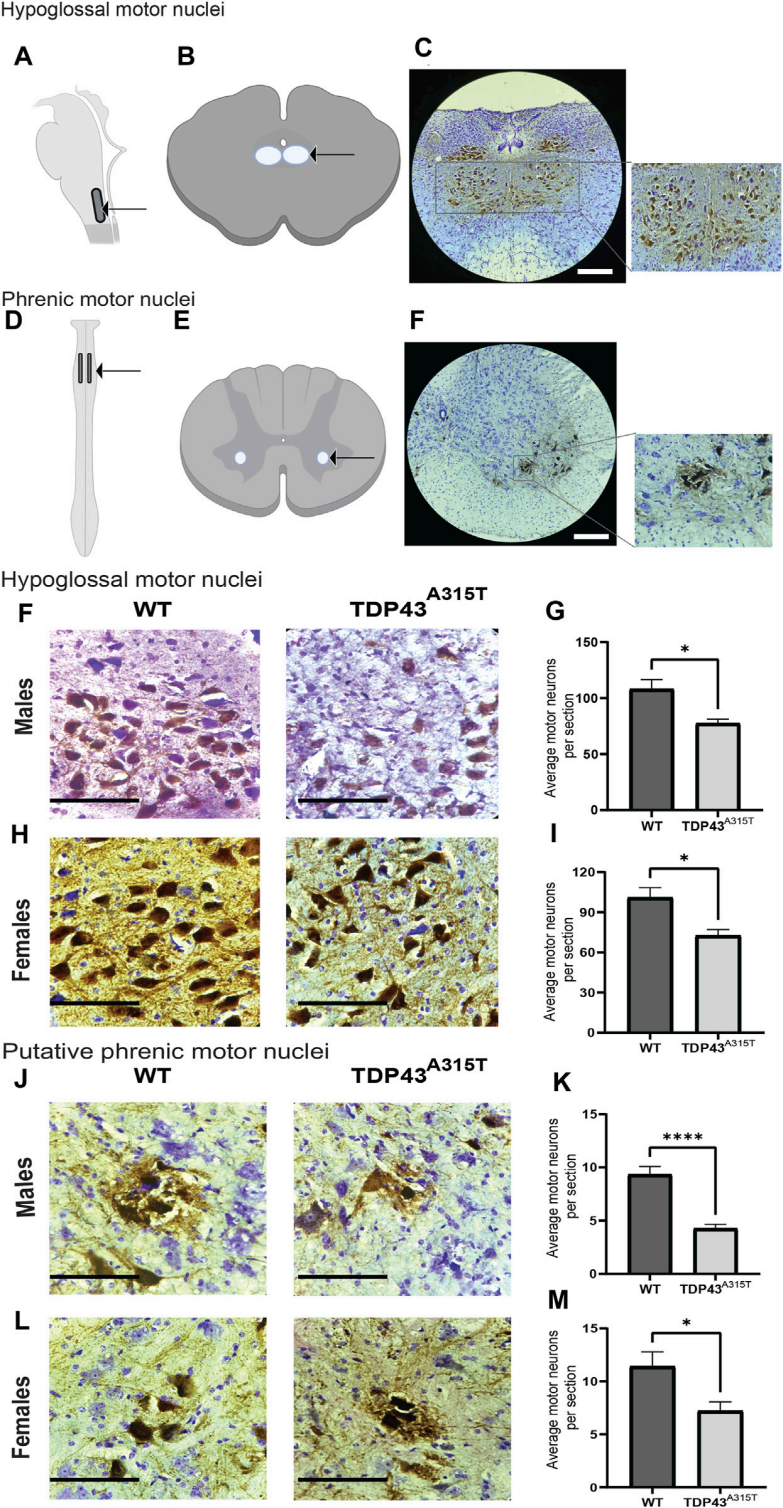
TDP-43^A315T^ mice exhibit neurodegeneration in the hypoglossal and putative phrenic motor nuclei. **(A,C)** Description of the location of hypoglossal motor nuclei. Schematic presentation of hypoglossal motor nuclei in the medulla **(A)** and transverse section of the medulla **(B)** shown with an arrow. Representative images of hypoglossal motor neurons (ChAT^+^) and creysl violet from male wild-type mice at 4X magnification. Scale bar = 890 μm **(C)**. **(D–F)** Description of the location of phrenic motor nuclei. Schematic presentation of phrenic motor nuclei in the cervical spinal cord (C3-C5 region) **(D)** and transverse section of the cervical spinal cord **(E)** shown with an arrow. Representative images of phrenic motor neurons (ChAT^+^) and cresyl violet from the C4 region in the cervical spinal cord of male wild-type mice at 4X magnification. Scale bar = 890 μm **(F)**. **(G,I)** Representative images of hypoglossal motor neurons (ChAT^+^) and creysl violet from male **(G)** and female **(I)** wildtype and TDP-43^A315T^ mice. Quantification of motor neurons from male **(H)** and female **(J)** hypoglossal motor nuclei of wildtype and TDP-43^A315T^ mice. (n = 4/genotype). **(K,M)** Representative images of putative phrenic motor neurons (ChAT^+^) creysl violet from male **(K)** and female **(M)** wildtype and TDP-43^A315T^ mice. Quantification of motor neurons from male **(L)** and female **(N)** putative phrenic motor nuclei of wildtype and TDP-43^A315T^ mice. (n = 4/genotype). Data are mean ± SEM. Statistical significance was determined using an unpaired Student’s T-test (*P < 0.05; **P < 0.01; ***P < 0.001). Scale bar = 90 μm.

To determine if respiratory insufficiency is associated with neurodegeneration, we investigated the histopathology of the motor neurons in the hypoglossal and putative phrenic nuclei in male and female WT and TDP43^A315T^ mice at end-stage. Hypoglossal motor neuron pools are located in the medulla (Figures 4A, B) while phrenic motor neuron pools are present in the cervical spinal cord (C3-C5) (Figures 4D, E). The location of the hypoglossal motor neuron pool (Figure 4C) and phrenic motor neuron pool (Figure 4F) are shown using representative images from WT mice. We used ChAT antibodies to stain the motor neurons in the medulla and cervical spinal cord (C3-C5 region) to visualize the hypoglossal (Figures 4G, I) and putative phrenic (Figures 4K, M) motor neuron pools, respectively. Compared to WT mice, both male and female TDP43^A315T^ mice have a significant decrease in both the hypoglossal (Figures 4H, J; *P =* 0.011 for male and *P =* 0.02 for female) and putative phrenic (Figures 4L, N; *P < 0.0001* for male and *P =* 0.04 for female) motor neurons.

**FIGURE 5:**
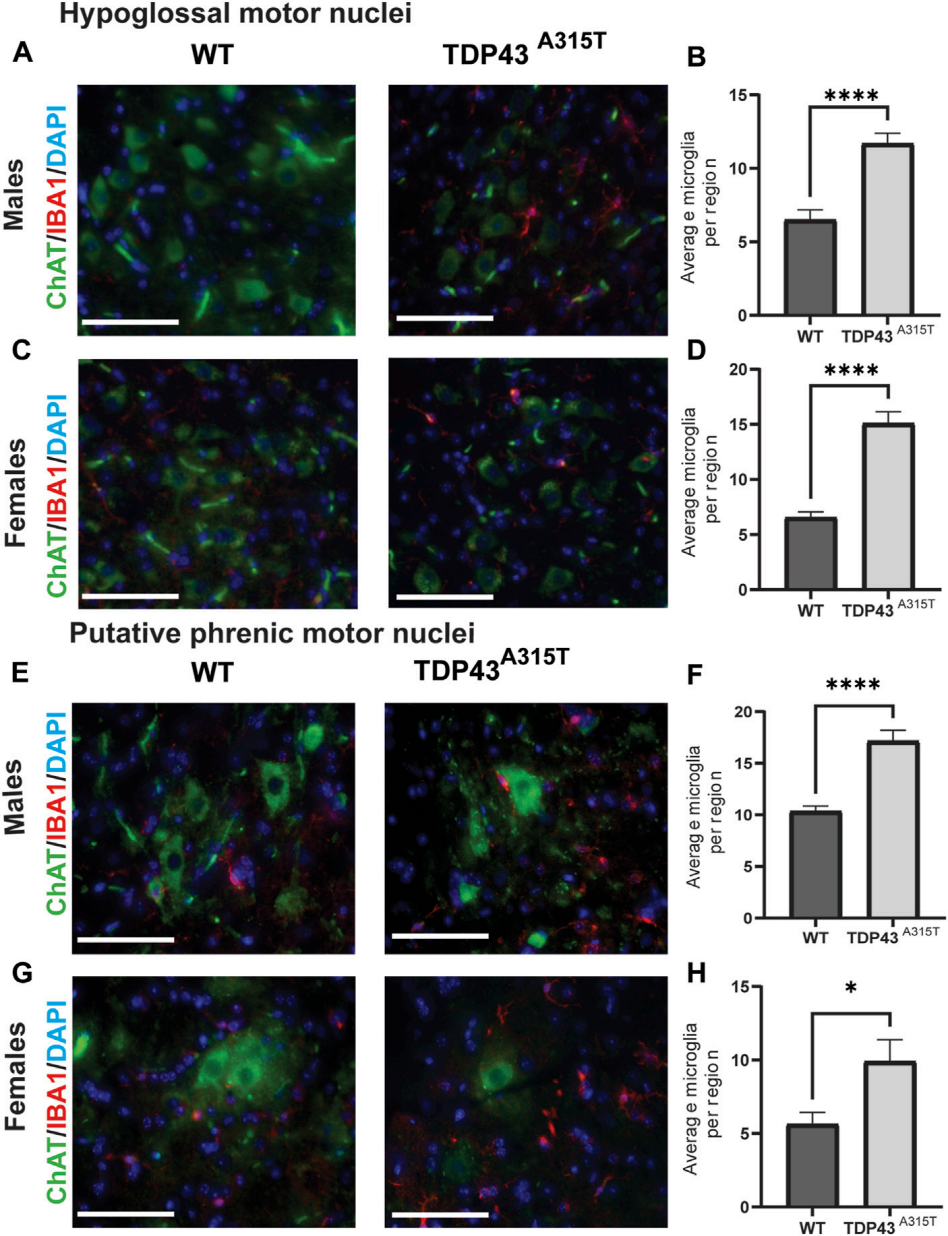
Increased number of activated microglia in the hypoglossal and putative phrenic motor nuclei of the TDP-43^A315T^ mice **(A–D)** Representative images of ChAT^+^ (green), Iba1^+^ (microglia) and DAPI (blue) stained hypoglossal motor nuclei in the medulla of male **(A)** and female **(C)** wildtype and TDP-43 ^*A315T*^ mice. Quantification of Iba1^+^ microglia from male **(B)** and female **(D)** hypoglossal motor nuclei of wildtype and TDP-43 ^*A315T*^ mice. (n = 4/genotype). **(E–H)** Representative images of ChAT^+^ (green), Iba1^+^ (microglia) and DAPI (blue) stained putative phrenic motor nuclei in the cervical spinal cord of male **(E)** and female **(G)** wildtype and TDP-43 ^*A315T*^ mice. Quantification of Iba1^+^ microglia from male **(F)** and female **(H)** putative phrenic motor nuclei of wildtype and TDP-43 ^*A315T*^ mice. (n = 4/genotype). Data are mean ± SEM. Statistical significance was determined using an unpaired Student’s T-test (*P < 0.05; ****P < 0.0001). Scale bar = 40 µm.

**FIGURE 6:**
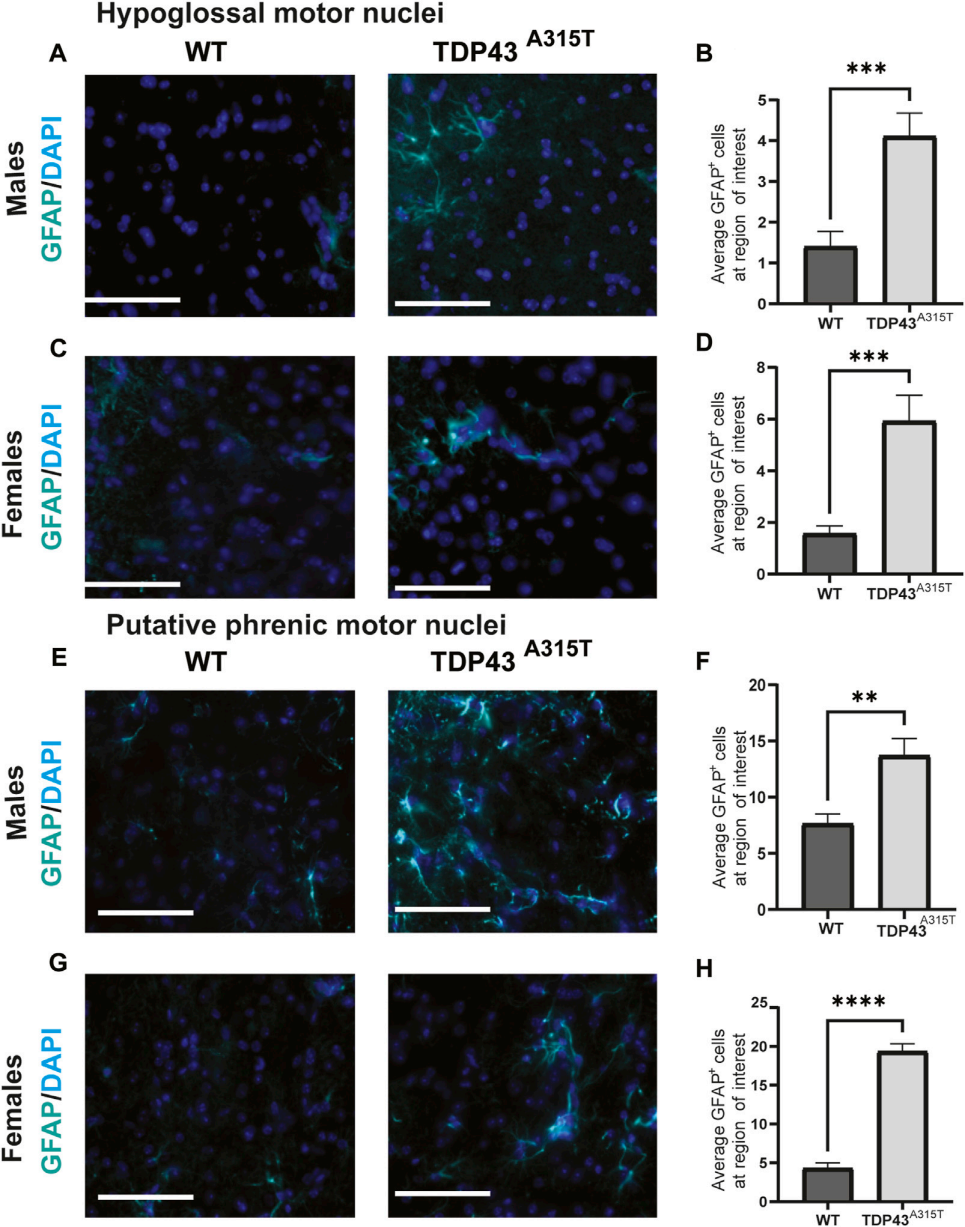
Increased astrocyte activation in the hypoglossal and putative phrenic motor nuclei of the TDP-43^A315T^ mice **(A–D)** Representative images of GFAP^+^ (astrocytes) and DAPI (blue) stained hypoglossal motor nuclei in the medulla of male **(A)** and female **(C)** wildtype and TDP-43^A315T^ mice. Quantification of GFAP^+^ astrocytes from male **(B)** and female **(D)** hypoglossal motor nuclei of wildtype and TDP-43^A315T^ mice. (n = 4/genotype). **(E–H)** Representative images of GFAP^+^ astrocytes and DAPI (blue) stained putative phrenic motor nuclei in the cervical spinal cord ofmale **(E)** and female **(G)** wildtype and TDP-43^A315T^ mice. Quantification of GFAP^+^ astrocytes from male **(F)** and female **(H)** putative phrenic motor nuclei of wildtype and TDP-43^A315T^ mice. (n = 4/genotype). Data are mean ± SEM. Statistical significance was determined using an unpaired Student’s T-test (**P < 0.01; ***P < 0.001 ****P < 0.0001) Scale bar = 40 μm.

To investigate neuroinflammation, we assessed the activation of microglia (Figure 5) and astrocytes (Figure 6) in the hypoglossal and putative phrenic nuclei in end-stage TDP43^A315T^ and age-, sex-matched WT mice. To evaluate the activation of microglia, we used immunohistochemistry staining of the medulla (Figures 5A, C) and cervical spinal cord tissues (Figures 5E, G) of both male and female WT and TDP43^A315T^ mice with anti-ChAT (to label for motor neurons) and anti-IBA1 (to label for microglia). We observed activated, ameboid-like microglia morphology of IBA1-positive cells in and around the hypoglossal and putative phrenic motor neuron pools of TDP43^A315T^ mice. Quantification of these Iba1+ cells reveals a statistically significant increase of activated microglia in both hypoglossal (Figure 5B, D; *P < 0.0001* for male and *P < 0.0001* for female) and putative phrenic (Figures 5F, H; *P < 0.0001* for male and *p = 0.0318* for female) nuclei. In addition, to evaluate astrogliosis, anti-GFAP (to label for astrocytes) was used in the medulla and cervical spinal cord (C3-C5 region) of WT and TDP43^A315T^ mice to visualize activated astrocytes in the hypoglossal (Figures 6A, C) and putative phrenic (Figures 6E, G) nuclei. When quantified, the number of activated astrocytes in and around the hypoglossal (Figures 6B, D; *p = 0.0003* for males and *p = 0.003* for females) and putative phrenic (Figures 6F,H; *p = 0.0022* for male and *P < 0.0001* for females) motor neuron pools were significantly elevated in TDP43^A315T^ mice.

### 3.4 Axonopathy in TDP-43^A315T^ mice

**FIGURE 7:**
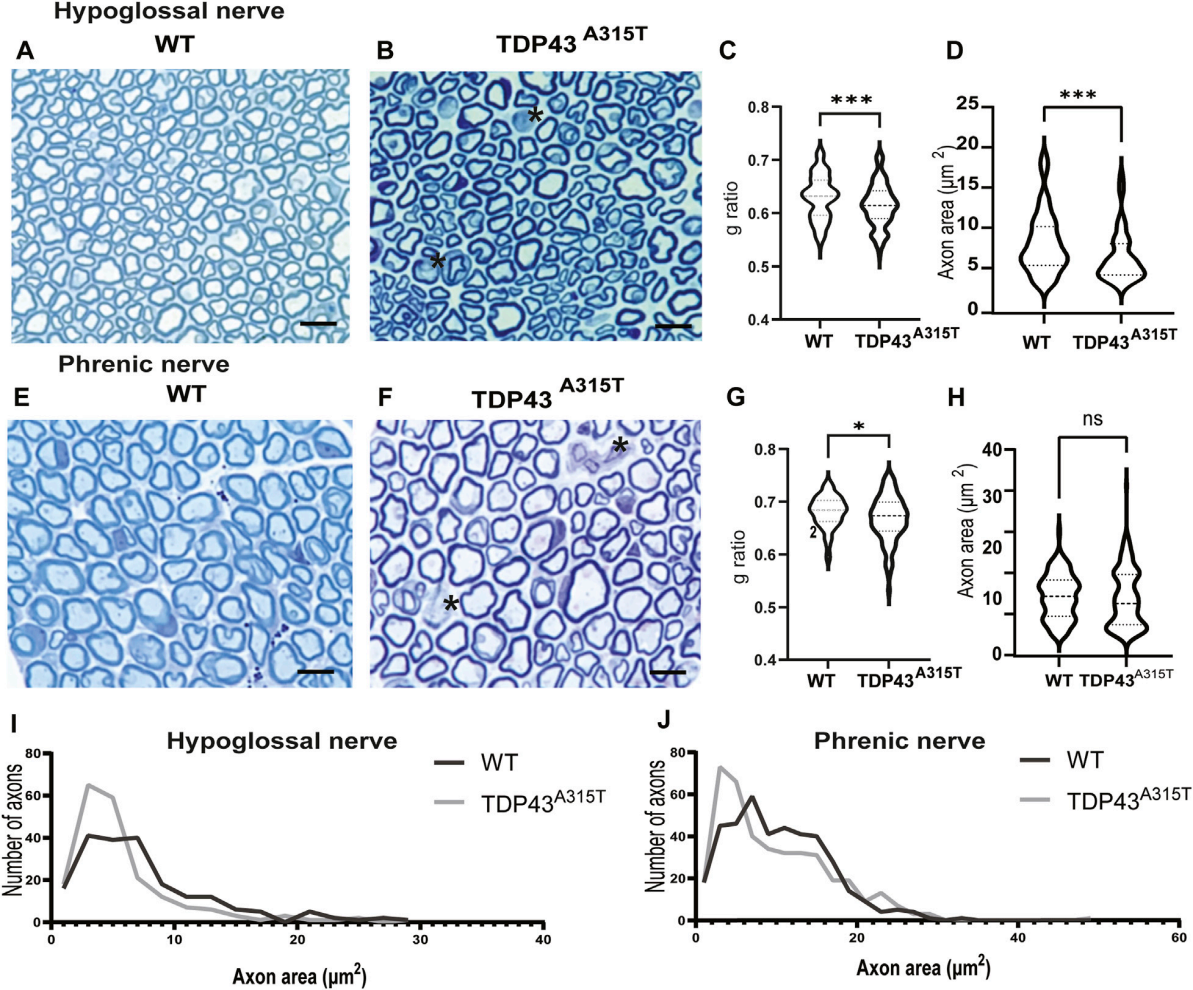
Axonopthy in TDP-43^A315T^ hypoglossal and phrenic nerve representative images of WT and TDP-43 ^*A315T*^ hypoglossal **(A,B)** and phrenic **(E,F)** nerve cross sections stained with toluidine blue from WT and TDP-43 ^*A315T*^ male mice. The asterisk indicates the demyelination of axons. Scale bar = 45 µm. **(C,F)** Quantification of g ratio of hypoglossal **(C)** and phrenic **(G)** axons from WT and TDP43 ^*A315T*^ male mice. **(D,H)** Quantification of area of axons in hypoglossal **(D)** and phrenic **(H)** nerves. **(I,J)** Distribution of axons relative to their size in hypoglossal **(I)** and phrenic nerve **(J)**. (n = 2/genotype).

To identify if respiratory dysfunction is associated with axonopathy, we studied the hypoglossal and phrenic nerve of male WT and TDP-43^A315T^ mice (Figure 7). Toluidine blue stained hypoglossal (Figures 7A, B) and phrenic (Figures 7E, F) nerves of TDP43^A315T^ mice show a decrease in myelin thickness than WT mice at their end stage (indicated by the asterisk). G-ratio is the ratio of the inner axonal diameter to the total outer diameter. It is widely used as a functional and structural index of optimal axonal myelination axons (Dobrowolny et al., 2015; Dhindsa et al., 2020; McCall et al., 2020; Shackleford et al., 2022). The g-ratio of hypoglossal (Figure 7C; *P < 0.0003*) and phrenic (Figure 7G; *P = 0.014*) nerves from TDP-43^A315T^ mice was significantly lower than WT mice. In addition, we found that the axonal area of the hypoglossal nerve is significantly reduced in TDP-43^A315T^ mice compared to WT (Figure 7D; *P* < 0.0006), and the distribution of the axon relative to their area showed that the hypoglossal nerve in TDP-43^A315T^ mice has lower number of smaller sized axons than WT mice (Figure 7I) similar to that of phrenic nerve (Figure 7J). In contrast, no differences were found in the axonal area of the phrenic nerve (Figure 7H; *P* = 0.4)

### 3.5 TDP43^A315T^ mice display an increased motor and behavioral disorder

**FIGURE 8:**
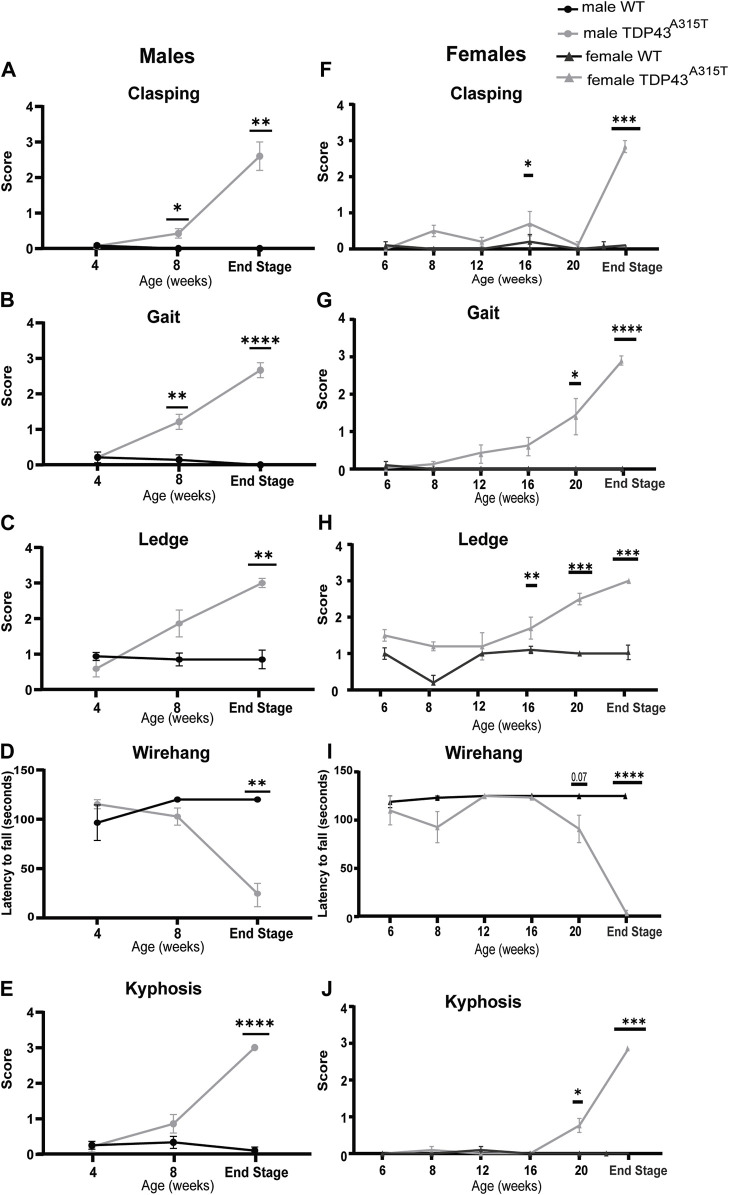
Increased motor and behavioral disorder in TDP-43^A315T^ mice at the end-stage. Behavioral analysis for both males **(A–E)** and female **(F–J)** WT and TDP-43^A315T^ mice for hind-limb clasping **(A,F)**, gait **(B,G)**, ledge tests **(C–H)** latency to fall during wire-hanging tests **(D,I)** and kyphosis **(E,J)** at indicated ages for males. Data are mean ± SEM. Statistical significance was determined using a mixed-model two-way ANOVA, followed by a Fisher’s LSD test (*P < 0.05; **P < 0.01; ***P < 0.001; ***P < 0.001).

Neurobehavioral assessments, including clasping, gait, kyphosis, ledge, and wire hang tests were performed to track the onset and worsening of symptoms in TDP-43^A315T^ mice (Figure 8). The ledge and gait tests are direct measures of coordination and evaluate the global sensorimotor functions in different mouse models (Pryor et al., 2014; Fusco et al., 2021). The clasping test is a marker of disease progression in neurodegenerative mouse models (Barbosa et al., 2022). Kyphosis, dorsal curvature of the spine, is a sign of muscle weakness (Guyenet S. J. et al., 2010), and the wire hang test measures muscle strength and neuromuscular function (Bellantuono et al., 2020; Barbosa et al., 2022).

At 4 weeks of age, no neurobehavioral differences are seen between male TDP-43^A315T^ and wild-type mice. Beginning at 8 weeks, male TDP-43^A315T^ mice earn significantly higher scores for the clasping and gait test. However, at the end-stage, the male TDP-43^A315T^ mice showed a significantly higher score than WT littermates in clasping (Figure 8A; *P* = 0.003), gait (Figure 8B; *P* < 0.0001), ledge test (Figure 8C; *P* = 0.004), wire hang (Figure 8D; *P* = 0.002) and kyphosis (Figure 8E; *P* < 0.0001) tests compared to male wild-type age-matched littermates.

Unlike males, the female TDP-43^A315T^ does not demonstrate a significant difference in clasping behavior until the end-stage (Figure 8F; *P* = 0.0003). However, these mice show a steady increase in other test scores with age and progression of disease compared to their WT female littermate. Similar to the males, female Prp-TDP-43^A315T^ mice earned the highest scores at their end-stage for gait (Figure 8G; *P* < 0.0001), ledge (Figure 8H; *P* = 0.0002), wire hang tests (Figure 8I; *P* = 0.0001) and kyphosis (Figure 8J; *P* < 0.001). Together, the weight and behavioral tests reveal that TDP-43^A315T^ mice display a motor phenotype including progressive loss of motor coordination and functions and muscle weakness.

## 4 Discussion

The primary findings of this study are that TDP-43^A315T^ mice have significant respiratory motor unit pathology and impaired respiratory function. These mice display sexual dimorphism in the onset of respiratory dysfunction, motor coordination, strength (disease onset), and survival. Respiratory deficits in awake, unrestrained, spontaneously breathing mice are evident by 8 weeks of age in males and 20 weeks in females, and these deficits become significantly worse at end-stage disease. Further, these mice have respiratory motor neuron pathology with a significant loss of hypoglossal and phrenic motor neurons, a dramatic increase of microglial and astrocyte activation in hypoglossal and phrenic motor nuclei, as well as hypoglossal and phrenic nerve pathology.

### 4.1 Respiratory dysfunction in ALS

ALS is a fatal neurodegenerative disease that leads to progressive paralysis and respiratory failure. Irrespective of whether the onset of ALS is bulbar or spinal, respiratory failure is the primary cause of death in patients with ALS (Tankersley et al., 1985; Schiffman and Belsh, 1993; Kleopa et al., 1999; Lyall et al., 2001; Al-Chalabi and Hardiman, 2013; Valko and Ciesla, 2019). TDP-43 pathology results in toxic accumulation in neurons of patients with both sporadic and familial forms of ALS (Neumann et al., 2006). Patients with TDP-43 A315T mutation have a slower progression of disease with progressive respiratory weakness, and eventual respiratory failure (Gitcho et al., 2008). To our knowledge, this is the first study to examine the respiratory motor unit pathology as a result of the TDP-43^A315T^ mutation in this mouse model. TDP-43^A315T^ mice have significant respiratory behavioural deficits which is exacerbated at the disease end-stage. Although there are not many studies of respiratory function in mouse models of ALS, we found that the TDP-43^A315T^ had similar respiratory deficits to the 6-months-old *Optn* ^*−/−*^ and SOD1^G93A^ mice at end-stage disease (Tankersley et al., 1985; Romer et al., 2017; Stoica et al., 2017; McCall et al., 2020). Specifically, SOD1^G93A^ mouse mice have significant respiratory pathology at 18 weeks of life and display a substantial drop off in TV, MV, PIF, and PEF by the time they reach the disease end-stage (Romer et al., 2017; Stoica et al., 2017). Similarly, *Optn* ^*−/−*^ mice, have a substantially reduced TV, MV, PIF, and PEF at 6 months of age, but this model was not as severe as the other models (McCall et al., 2020). In our studies, we found differences in MV, TV, PIF, and PEF in both male and female TDP-43^A315T^ compared to WT age and sex-matched mice. Female TDP-43^A315T^ mice have no significant differences from control at baseline, but show deficits when challenged. On the other hand, male TDP-43^A315T^ mice have a significant decline in F, TV, MV and PEF at baseline and these differences became much more robust during the respiratory challenge.

Gender-specific ALS onset and progression of the disease have been previously reported in pre-clinical models as well as in humans (Stoica et al., 2017). Men are more likely to develop ALS at earlier ages than women. However, the risk of onset of ALS increases with age in both sexes. The gender-specific differences in ALS are governed by various factors such as hormones, age, genetics, environment, and physical activity. These differences are evident in ALS patients (McCombe and Henderson, 2010; Hou et al., 2019; Tang et al., 2019; Zamani et al., 2024) and ALS SOD1 mouse models (Heiman-Patterson et al., 2005; Alves et al., 2011; Pfohl et al., 2015). Longer exposure to female reproductive hormones such as estrogen has a neuroprotective effect on motor neurons in ALS (de Jong et al., 2013; Blasco et al., 2012; Raymond et al., 2021; Trojsi et al., 2020). Treatment of 17α-estradiol in primary cultures of rat spinal cord can prevent glutamate- and nitric oxide (NO)-induced selective motor neuron death (Nakamizo et al., 2000). In addition, 17β-estradiol treated male SOD1^G93A^ mice displayed an increase in the survival of motoneurons, reduced NLRP3 inflammasome activation, and enhanced motor function (Heitzer et al., 2017). Another female hormone, progesterone which is expressed in the brain, spinal cord, and peripheral nervous can activate autophagy in the astrocytes of the spinal cord and can further delay the neurodegenerative process in SOD1^G93A^ transgenic mice (Kim et al., 2013). ALS patients showing higher testosterone levels and lower progesterone/free testosterone ratio presented a more rapid worsening of the respiratory parameters (Gargiulo-Monachelli et al., 2014). Exposure to androgens in SOD1^G93A^ ALS mice induces muscle hypertrophy, reduces the motor neurons and exacerbates the disease phenotype (Aggarwal et al., 2014). ALS in female patients predominantly occurs in the post-menopausal phase whereas the female mice in this study were pre-menopausal. However, they displayed a later onset of disease. We speculate that since a longer exposure to female reproductive hormones has a neuroprotective effect on motor neurons that such differences in hormones can be responsible for the sex-specific difference in the onset and progression of the disease in the TDP-43^A315T^mice. Further examination of the hormonal effects on respiratory pathology is needed in the TDP-43^A315T^ mouse model.

No previous studies have reported respiratory insufficiency due to mutation in the TDP-43 protein. Compared to other TDP-43 transgenic mice models, such as TDP-43^Q331K^, TDP43^A315T^ mice exhibit striking differences in sex-specific differences (Wong et al., 2020; Watkins et al., 2021). One of the groups has reported that female TDP-43^Q331K^ knock-in mice have less severe progressive behavioral phenotypes than male mutants (White et al., 2018; Watkins et al., 2020) Further studies are required to understand how a mutation in TDP-43 can manifest different timelines for the onset, progression and severity of disease symptoms including respiratory dysfunction.

### 4.2 Neuropathology in ALS-associated respiration

Prior studies showed that the mice with A315T mutation in TDP43 develop motor neuron disease and exhibit degeneration of axons and loss of neurons (Wegorzewska et al., 2009; Ke et al., 2015). Thoracic spinal cords from TDP43^A315T^ mice had less number of axons with numerous degenerating axons in both the dorsal corticospinal tract and lateral columns (Wegorzewska et al., 2009). Using another mouse model with A315T mutation in TDP43, pronounced atrophy was observed in the hippocampus and cortical regions with reduced TDP-43^*A315T*^ expressing neurons within the CA1 region of the hippocampus (Ke et al., 2015). Our focus was on the respiratory control centers of the hypoglossal and phrenic motor nucleus. Specifically, we sought to define the extent of neuropathology motor neuron loss and neuroinflammation in the hypoglossal and phrenic respiratory control centers in the TDP-43^A315T^ mice. Hypoglossal motor neurons in the medulla innervate the tongue and maintain the upper airway patency while the phrenic motor neurons in C3-C5 regions of the cervical spinal cord innervate the diaphragm, which is a major inspiratory muscle. The loss of hypoglossal and phrenic motor neurons is observed in end-stage SOD1^G93A^ mice (Haenggeli and Kato, 2002; Ferrucci et al., 2010) and rats (Lladó et al., 2006; Nichols et al., 2015). The loss of hypoglossal motor neurons and reduction in its size was also observed in a different strain of SOD1 mutant G86R superoxide dismutase transgenic mice (Nimchinsky et al., 2000). In addition, Optn^−/−^ mice displayed loss of hypoglossal motor neurons at 12 months of age (McCall et al., 2020). Here, we observed a drastic loss of both hypoglossal and phrenic motor neurons in male and female TDP-43^A315T^ mice at the disease end-stage which could contribute to the respiratory deficits found.

The TDP-43^A315T^ mice, especially females demonstrated a significant difference in breathing during hypoxic and hypercapnic challenges compared to wild type mice.

A315T mutation in TDP-43 impacts glial cells and motor neurons in the respiratory motor nucleus. In addition, it contributes to hypoglossal and phrenic pathology. It can be speculated that significant pathology in motor neurons and nerves can impact the respiratory muscles causing respiratory disorder when stressed with hypoxic and hypercapnic challenges.

The pre-Bötzinger complex (pre-BötC) in the medulla is responsible for the generation and regulation of the respiratory rhythm (Smith et al., 1991). The activation of hypoglossal motor neurons is influenced by the excitation of pre-BötC neurons to maintain upper airway patency during breathing (Muñoz-Ortiz et al., 2019). The central respiratory chemoreceptor (CRC) cells are sensitive to changes in pH or PCO_2_ in the central nervous system. These changes can further influence the frequency of breathing during hypercapnia and hypoxia (Guyenet P. G. et al., 2010). Further studies are required to analyze the impact of A315T mutation in TDP-43 in these regions and how they contribute during hypoxic and hypercapnic environments. In addition, the impact of the respiratory centers in the hypothalamus and limbic system needs to be further investigated.

In addition to neurodegeneration, neuroinflammation is a distinctive feature of ALS (Liu and Wang, 2017; Ban et al., 2019; Tam et al., 2019; You et al., 2023). Specifically, an increase in activation of microglia is observed in the corticospinal tract of ALS patients (Brettschneider et al., 2012). Proinflammatory microglia in SOD1^G93A^ mice produce reactive oxygen species and nitric oxide and decrease the production of neurotrophic factors resulting in motor neuron cell death causing the progression of diseases (Beers et al., 2006; Xiao et al., 2007; Frakes et al., 2014). Activation of astrocytes also plays a critical role in the progression of ALS symptoms and reducing reactive astrocytes has resulted in slow progression of disease in SOD1 mice (Clement et al., 2003; Wang et al., 2011). Astrocytes expressing A315T mutation in TDP-43 mice trigger motor neuron cell death mediated via sodium channels and nitroxidative stress (Rojas et al., 2014). We observed a notable increase in Iba1^+^ microglia and GFAP^+^-activated astrocytes in the hypoglossal and phrenic motor control centers of both male and female TDP-43 mice. These activated glia can create a proinflammatory environment at respiratory control centers which can further be responsible for the death of motor neurons.

Lack of functional TDP-43 in astrocytes results in the formation of A1-like proinflammatory astrocytes activating microglia and reducing the number of mature oligodendrocytes (Peng et al., 2020). Glia such as oligodendrocytes and Schwann cells produce myelin. We observed a significant decrease in the g-ratio and reduced thickness of myelin in hypoglossal and phrenic nerves in TDP-43^A315T^ mice. Loss of oligodendrocytes and Schwann cells or accumulation of pathological TDP-43 protein in glia may explain demyelination in TDP-43 mice, however, future studies need to confirm this (Nakamura-Shindo et al., 2020).

In addition, we observed that TDP-43^A315T^ mice displayed reduced myelin thickness in both hypoglossal and phrenic axons. Also, a significant reduction of the hypoglossal axonal area was recorded in TDP-43^A315T^ mice compared to WT mice. In contrast, no differences were observed in the phrenic axonal area. However, a skewed distribution favoring smaller axons was observed in both hypoglossal and phrenic nerves. Typically nerves from wildtype mice are comprised of diverse-sized axons for normal muscle functions. Small axons convey intense information signals to a small number of muscle fibers, whereas large axons transmit less intense information signals to a larger number of muscle fibers (Perge et al., 2012). The consequences of the different hypoglossal and phrenic axonal areas in breathing deficits of TDP-43^A315T^ mice need to be further investigated.

The majority of TDP-43 proteins in motor neurons are present in the nuclei and a population of the protein is found in highly mobile granules that are actively transported along the axons of the motor neurons (Fallini et al., 2012). The gain or loss of the TDP-43 function can instigate its mislocalization and also reduce the dendritic complexity caused by the suppression of dendritic growth (Herzog et al., 2017; Dyer et al., 2021). Based on this information, we speculate that the mutation in TDP-43 can potentially affect the formation or durability of excitatory synapses within the respiratory neural network contributing to the erratic breathing observed in TDP-43^A315T^ mice and can be further explored.

### 4.3 Limitations of this study and other complications in TDP-43^A315T^ mice

According to prior studies, TDP-43^A315T^ mice are prone to developing gastrointestinal (GI) complications, which can lead to intestinal dysmotility and contribute to sudden death in these mice (Esmaeili et al., 2013; Hatzipetros et al., 2014; Herdewyn et al., 2014). Interestingly, these GI complications were not observed in other ALS mouse models, indicating that the TDP-43^A315T^ mice may have a unique disease phenotype.

Abdominal distention can affect breathing limiting diaphragm motility and thereby causing restrictive lung disease. This can result in a decreased tidal volume and a possible decrease in minute ventilation but would not decrease the frequency. There was a significant decrease in TV, PIF, and PEF in TDP-43^A315T^ mice, which suggests weakness of the inspiratory muscles and expiratory muscles (Huang et al., 2011). The GI complications may be a possible contributing factor to these findings of respiratory phenotype and further studies would need to tease out the contribution of GI pathology to these respiratory findings. However, the neurodegeneration and neuroinflammation in the respiratory control centers of TDP-43 support the hypothesis that there is also significant respiratory motor neuron pathology contributing to these respiratory deficits.

## 5 Conclusion

In conclusion, the TDP-43^A315T^ mice have an early onset, rapid progression of disease, and premature death. These mice have profound breathing deficits at baseline and during a respiratory challenge. They also have neurodegeneration and neuroinflammation in the hypoglossal and putative phrenic respiratory motor nuclei as well as demyelination of the hypoglossal and phrenic nerves. Respiratory involvement and progressive respiratory weakness have been reported in patients with the A315T mutation in TDP-43, and this study confirms that this mutation results in significant neuropathology in the hypoglossal and phrenic motor units as well as respiratory deficits. Future studies that investigate TDP-43 mutations’ role in chemosensitivity, muscle weakness, and disruption of neuromuscular junctions will further clarify underlying mechanisms that result in respiratory pathophysiology.

## Data Availability

The raw data supporting the conclusions of this article will be made available by the authors, without undue reservation.
